# Telomere length as a biomarker for cumulative experience in broiler chickens

**DOI:** 10.1371/journal.pone.0326195

**Published:** 2025-06-25

**Authors:** Andrew M. Campbell, Mallory G. Anderson, Mark F. Haussmann, Raquel Rowell, Leonie Jacobs

**Affiliations:** 1 Virginia Tech, School of Animal Sciences, Blacksburg, United States of America; 2 Bucknell University, Department of Biology, Lewisburg, United States of America; University of Newcastle, UNITED KINGDOM OF GREAT BRITAIN AND NORTHERN IRELAND

## Abstract

Cumulative experience can be defined as the sum of all positive and negative experiences during an animal’s lifetime. Telomere length shows promise as a biomarker of cumulative experience in humans and non-human animals but is not yet assessed for broiler chickens. Therefore, our objective was to determine telomere length changes due to positive and negative experiences in fast-growing broiler chickens. In three replicated experiments, male Ross 708 broilers were housed in a 2 × 2 factorial study investigating high environmental complexity as a positive environment (vs. low complexity; 6 pens/treatment) and high stocking density as a negative environment (vs. low density; 6 pens/treatment). Telomere length was quantified at day 48 of age via quantitative RT-PCR (qRT-PCR) from gonad and kidney samples (N = 9 samples/treatment/tissue/experiment). Prior to analysis, raw relative telomere length (rTL) values were z-transformed to allow comparison between experiments. Combined data from the three experiments were analyzed using mixed models with complexity, density, and their interactions as fixed factor and pen nested within experiment and qRT-PCR plate number as random factors. Over all three trials, birds housed in high complexity environments tended (P = 0.0503) to have longer telomeres from kidney tissue than birds housed in low complexity environments. Stocking density did not impact combined kidney telomere length and gonadal telomere length was not impacted by environmental complexity or stocking density. Longer telomeres (statistical trend) in response to positive experience (environmental complexity) when compared to low-complexity indicate that high-complexity environments elicited positive cumulative experience in broiler chickens, although effect size was small. Telomere length has the potential to be a valuable tool in the assessment of cumulative experience in production settings, and future works should replicate these findings and expand upon this work by comparing telomere length with other more traditional animal welfare markers.

## Introduction

Ethical concerns over production animal treatment highlight our responsibility to optimize animal welfare outcomes. This includes ensuring that production animals have healthy biological functioning, the ability to display natural behaviors, and experience positive emotional states [1]. Animals in our care undergo positive and negative events which contribute to their cumulative welfare status [2,3]. Therefore, the term ‘cumulative experience’ is used to refer to the total sum of all positive and negative impacts on the health, behavior, and emotional states of production animals during their lifetime [2,4]. High stocking density is a negative condition, while environmental complexity is a positive condition, and together these can both impact cumulative experience in broilers [5–7]. However, this impact is difficult to quantify as no measures of cumulative experience exist for broiler chickens. What measures of animal experience do exist mainly focus on negative experience and affect, such as fear, distress, and anxiety [8–11]. As nearly all measures of broiler experience rely on interpretations of behavior, a physiological measure of cumulative experience in broiler chickens could be valuable to the poultry industry.

Recently, telomere length has gained attention as a potential marker of cumulative experience in humans [12–16], laboratory animals [17–19], poultry [20–22] and wild animals [23–28]. Telomeres are protein-DNA complexes which end-cap chromosomes and protect gene-encoding DNA from the ‘end-chain replication problem [2]. Telomeres shorten every time a cell divides, which is a primary contributor to cellular aging and apoptosis in somatic cells [2,29]. These end-cap telomeres protect against cellular aging and apoptosis. However, telomeres do not only shorten during mitosis, but also in response to oxidative stress [2,30]. Associations between aging and stress have long been established [31–38] and have led to a distinction between chronological age and biological age [2,39]. In the context of telomeric shortening, or telomeric attrition, chronological age is the time-proportional shortening of telomeres in response to the number of mitotic divisions of a cell. Biological age is the combination of chronological age and changes in telomeric attrition caused by lifetime events.

Telomere length in humans has been proposed as a ‘psychobiomarker’ of cumulative lifetime stress and negative experience [2,40]. For example, shorter telomeres and increased biological age have been associated with anxiety [12], depression [13], neuroticism [16], pessimism [15], family disruption [41], chronic pain [42,43], and childhood exposure to violence [44]. Additionally, telomere length in humans seems responsive to positive experience. Healthy lifestyle choices decrease the rate of telomere shortening in response to stressful life events [45] and increase concentrations of telomerase [13] which should decrease biological age. Telomere attrition was associated with aging in cattle, pigs, sheep and horses, although stressors were not the focus in these studies (for a review, see [46]). In dairy calves, pre-natal temperature-humidity conditions were associated with shorter telomeres [47]. Some associations between negative experiences and telomere length were reported in mice too. Telomere length in mice was negatively impacted by overcrowding [19], excessive reproduction [19], forced participation in stressful challenges [17], and salmonella infection [18]. Based on research in other species, plus the limited research done in poultry, telomere length shows promise for this measure as a biomarker for animal welfare. Telomere length was decreased in laying hens housed at high stocking density and after a 14-day feed restriction [21]. Broilers fed a diet with corticosterone showed shortened telomere lengths in whole blood, muscle, liver and heart samples [48] and shows a positive association with gastrointestinal pathologies under chronic stress [49]. Broilers housed in high-density environments showed decreased telomere length compared to birds housed in low-density environments, indicating an increased biological age in the former [20]. Thus, telomere length shows potential to determine cumulative experience and biological age in relation to housing conditions [20].

High stocking density is generally considered a negative housing stimulus, as broilers raised in high-density environments can show increased lameness [50,51], increased distress [20,52,53] and decreased ability to display natural behaviors [54,55]. Broilers are often raised in low-complexity environments that restrict broilers’ ability to display species-specific behaviors, lead to boredom and frustration, and negative affective states [56–59]. Broilers raised in low-complexity environments show increased fear and anxiety and decreased optimism compared to broilers raised in high-complexity environments [58,59], indicating that birds from low-complexity environments were in a worse affective state. The provision of environmental enrichment, thus a more complex environment, has positive impacts on broiler health [6,60,61], behavior [6], affective state [58], stress [62], and can make broilers more resistant to stressful events [62]. Reduced distress induced by environmental complexity could slow the rate of telomere shortening in those broilers. Additionally, the positive experience provided by environmental complexity could mirror the effects seen in humans where positive experience increased telomerase activity and reversed the impacts of stress on telomeric attrition [12,14,45,63,64].

The telomere restriction fragment (TRF) analysis has long been considered the ‘gold standard’ for telomere length quantification [65–69]. However, TRF is low throughput, while real-time quantitative polymerase chain reaction (qRT-PCR) assay has emerged as a suitable alternative to TRF for telomere length quantification [65,69–72]. However, qRT-PCR has only been used once to measure telomere length in domestic chickens [48] and therefore requires further validation via comparison with TRF analysis. Additionally, telomere dynamics differ between tissues [73], thus it is worth determining which tissues are suitable to sample. For example, gonadal tissue is primarily comprised of stem cells which produce a high amount of telomerase [74–76]. Telomerase is capable of directly adding new telomeric DNA repeats to chromosome ends so repairing telomeric DNA in vertebrates [77,78]. Telomerase is expressed in very low quantities in somatic cells and in high quantities in stem cells. Therefore, gonadal telomere length should not decrease due to cell division and should not decrease due to cumulative experience if telomerase activity is sufficient to maintain telomere length, however, this is not confirmed. In contrast, kidney cells are primarily somatic cells with low concentrations of telomerase [79]. In these cells, telomere length may be susceptible to cumulative experience. The impacts of environmental complexity and high stocking density on telomere length in broilers is mostly unknown [20]. Therefore, the objective of this study was to determine how telomere length is impacted by environmental complexity (positive experience) and high stocking density (negative experience) compared to opposing conditions in broiler chickens. We hypothesized that relative telomere length (rTL) values from broiler chicken gonadal tissue would be unaffected by experimental treatments. Additionally, we hypothesized that rTL values would be increased in broilers housed in high-complexity environments and decreased in birds housed in high-density environments compared to birds housed in low complexity and low density, respectively. Finally, we hypothesized that birds housed in high-complexity, low-density environments would show longer telomeres than birds housed in low-complexity, high-density environments.

## Methods

### Experimental design

All procedures in this study were approved by the Virginia Tech Institutional Animal Care and Use Committee (IACUC protocol 19–175). This study was designed using a 2 × 2 factorial approach with environmental complexity (high or low) and stocking density (high or low), resulting in four treatment groups: low-density/low-complexity, low-density/high-complexity, high-density/low- complexity, high-density/high-complexity.

### Birds and housing

In three replicated experiments, 1-day-old male Ross 708 chicks (n = 1,620/experiment) were randomly sorted into four treatment groups, three replicate pens per treatment, with 12 pens per experiment. Experimental design is described in detail in [58,59]. The four treatments were randomly distributed over 12 pens (3 pens/treatment). Pens (14.5 m^2^) contained clean pine wood shavings, four galvanized steel feeders, and 3 nipple water lines (3 nipples/line). Birds had ad libitum access to feed and water and were phase fed a commercial corn/soy diet which was formulated to meet their nutritional needs. This diet included a starter (d1-d14), grower (d15-d28), and finisher phase (d28-d50). Pens contained three heat lamps and 24h continuous lighting during the first week of life, and 18h light:6h dark after the first week with a light intensity of 15 lux during light hours. In experiment 1, chicks unintendedly received 24h light for an extra week (until d14 of age). On d1, temperatures within the house were 35°C and gradually decreased to 21°C on d24, which was maintained until d50. In experiment 1, some birds were naturally infected with infectious bronchitis, which was unintended, thus not part of the experimental design. Once the infection was identified (morbidity approximated at 100%, mortality due to the pathogen was 3.6%), all birds received a therapeutic dose of antibiotics from d33 to d40 of age as prescribed by a veterinarian. Throughout the experiments, bird health and behavior were monitored twice a day.

### Environmental complexity

High-complexity pens were divided into four functional areas with permanent and temporary enrichments. These areas were a feeding area (3.2 m^2^) with four galvanized steel feeders and 1/3 of a mineral pecking stone broken into smaller pieces (Proteka, Inc., Lucknow, ON, Canada), a comfort area (3.2 m^2^) with a 2m^2^ wooden dust bath with playground sand, a play and exploration area (4.3m^2^) with paired sets of temporary enrichments, and a rest area (3.2 m^2^) containing three sets of perching structures. In experiment 1, PVC pipe perching structures (183 cm L × 31 cm W × 9 cm H × 1.9 cm diameter) provided 15.2 cm of linear perching space per bird in low-density pens and 7.6 cm in high-density pens. In experiments 2 and 3, wooden boards were provided as perching structures (122 cm L × 46 cm W × 8 cm H) and provided 76 cm^2^ of perching space in low-density pens and 39 cm^2^ of perching space in high-density pens. Temporary enrichments were grouped in pairs and rotated every 3d. Pairs were (1) hanging bundles of white polypropylene string and metal wire balls with alfalfa hay (Darice, Strongsville, OH, USA), (2) plastic balls (5.8 cm diameter; Click N Play, Bellevue, WA, USA) and treat dispensers (7.6 cm diameter; Lixit Corp., Napa, CA, USA) filled with oats, (3) red laser light (5 min; twice per day; Ethical Products, Inc., Bloomfield, NJ, USA) and Kong toy (5.6 cm diameter; KONG, Golden, CO, USA) with iceberg lettuce (experiment 1), or half a head of cabbage hung at bird height (experiment 2 and 3). The low-complexity pens were also divided into four areas but contained no enrichments. The four galvanized steel feeders were distributed in three areas of the pen.

### Stocking density

High-density pens contained 180 birds and low-density pens contained 90 birds to target a final stocking density of 40–42 kg/m^2^ in high-density pens and 20–22 kg/m^2^ in low-density pens. Final stocking densities at d50 were 42.1 kg/m^2^ (experiment 1), 42.6 kg/m^2^ (experiment 2), and 42.1 kg/m^2^ (experiment 3) in high-density pens and 23.8 kg/m^2^ (experiment 1), 23.3 kg/m^2^ (experiment 2), and 22.1 kg/m^2^ (experiment 3) in low-density pens.

### Measurements

**Fig 1:**
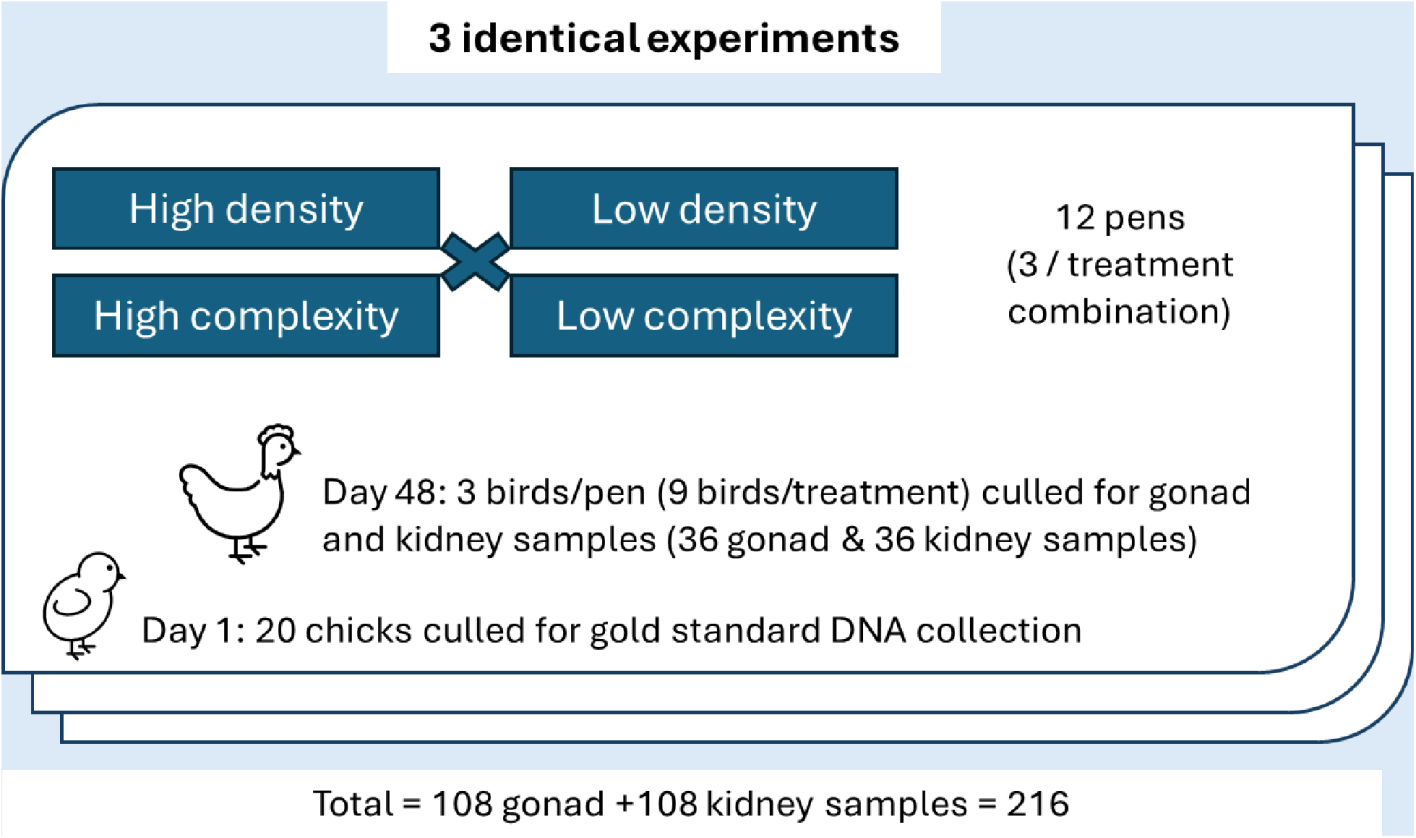
Visual representation of the 2 × 2 factorial experimental design, with an emphasis on sample sizes for relative telomere length analysis.

The methods in [80] were followed to determine rTL in the current study. Twenty birds per experiment were not assigned to a treatment group but were euthanized via cervical dislocation at d1 of age. Their kidneys and gonads were collected using macro-dissection to serve as gold standard DNA samples. These gold standard samples (“golden sample” in [80]) were pooled and used as reference DNA samples to calculate rTL in samples collected later in life. At d48 of age, three birds/pen were euthanized via cervical dislocation and kidney and gonad samples were collected using macro-dissection for quantification of telomere length. This resulted in 36 kidney and 36 gonad samples per experiment (9 samples/tissue/treatment), and a total of 216 samples for all three experiments (Fig 1).

Kidney and gonad samples were placed in 1.5 mL microcentrifuge tubes and immediately stored on dry ice before transport and storage at −80°C. Kidney and gonad samples were thawed and DNA was extracted using the QIAamp DNA mini kit (Qiagen, Hilden, Germany) following the manufacturer protocol. DNA purity was assessed as described by [80]. Purified DNA samples were then nano-dropped and diluted using molecular-grade water to standardize the amount of DNA in each sample (10 µg/µL). Following dilution, samples were stored at −80°C for up to three months prior to quantification and all samples underwent less than three freeze/thaw cycles. DNA integrity gels were used to ensure good quality DNA for qRT-PCR analysis, and all samples were of high quality as they yielded a single high molecular weight band. Relative telomere length was determined using an qRT-PCR method described [71,72]. In short, the proportion of telomeric DNA is calculated relative to the quantity of DNA from a single copy gene, glyceraldehyde-3-phosphate dehydrogenase (GAPDH), in the same DNA sample to form a ‘relative telomere length’ value, or rTL. This proportion reflects the length differences between telomeres and the constant GAPDH amplicon. Telomere primers were Tel1b 5’—CGG TTT GTT TGG GTT TGG GTT TGG GTT TGG GTT TGG GTT—3’ AND Tel2b 5—GGC TTG CCT TAC CCT TAC CCT TAC CCT TAC CCT TAC CCT –3’. The control GAPDH primers were GAPDHF 5’—CCT AGG ATA CAC AGA GGA CCA GGTT—3’ and GAPDHR 5’—GGT GGA GGA ATG GCT GTCA—3’. Samples were randomized and reactions were set up on 96-well plates (Qiagen) with a total reaction volume of 15 µL, containing 3.6 µL molecular grade water, 0.15 µL (1 µM) forward primer, 0.15 µL (1 µM) reverse primer, 7.5 µL SybrGreen (Qiagen), and 3.6 µL sample. PCR plates were covered using plastic adhesive plate covers and reactions were analyzed using a 7500 Fast Real-Time PCR machine (Applied Biosystems, Beverly, MA, USA). Eight plates were run, four each for kidney and gonad samples. A standard curve was created for each plate using the gold standard DNA extracted from the day-old chicks using the QIAamp DNA mini kit. These gold standard extractions were pooled and a two-fold serial dilution of 40, 20, 10, 5, and 2.5 ng/µL was used on each plate. All controls and samples were run in triplicate. All standard curves were used for quality control and all plates had standard curves within acceptable ranges with efficiencies within 100 ± 15 and R^2^ > 0.98. Individual well efficiencies and quantification cycle (C_q_) values were calculated in LinregPCR [80]. Relative TL was calculated using Equation 1. Individual well efficiencies were raised to the power of delta C_q_ (interplate control C_q_ minus individual sample C_q_; Equation 1). The resulting triplicate telomere and GAPDH values were then averaged and divided by each other to create a single rTL value (Equation 1) [71,72].


$$\begin{array}{c} \text{rTL}={\frac{{{\text{(E}}_{\text{telomere}})}^{\Delta {\text{Cq}}_{\text{telomere}}}}{{(\text{E}}_{\text{\{GAPDH}})}}^{\Delta {\text{Cq}}_{\text{GAPDH}}}\} \end{array}$$ (1)

Equation 1. Equation used to calculate relative telomere length (rTL) of kidney or gonad samples from broiler chickens. Using this equation, the individual well efficiencies (E) of telomere or GAPDH are raised to the power of the inter plate control C_q_– target C_q_ (delta C_q_). This equation is previously described in [72].

### Validation of qRT-PCR assay

The qRT-PCR assay for domestic chickens was validated by comparing the qRT-PCR measurements with measurements of the same samples quantified using a telomere restriction fragment (TRF) assay [81]. To determine correlations between qRT-PCR and TRF, a subset of samples (N = 17) was quantified using both assays. For the TRF assay, 10 µg quantity of DNA was digested using 1.0 µL of RsaI (R0167L, New England Biolabs, Ipswich, MA, USA) and 0.2 µL of HinfI (R0155M, New England Biolabs) in CutSmart Buffer (B7204S, New England Biolabs) overnight at 37°C. The digested DNA was separated using pulsed field gel electrophoresis (3 V/cm, 0.5- to 7.0-second switch times, 14C) for 19 hours on a 0.8% nondenaturing agarose gel. The gel was then dried without heating and hybridized overnight with a 32P-labeled oligo (50-CCCTAA-30) that binds to the 3’ overhang of telomeres. Hybridized gels were placed on a phosphor screen (Amersham Biosciences, Buckinghamshire, UK), which was scanned on a Storm 540 Variable Mode Imager (Amersham Biosciences). Densitometry (ImageQuant 5.03v and ImageJ 1.42q) was used to determine the position and strength of the radioactive signal in each of the lanes compared to the molecular marker (1 kb DNA Extension Ladder; Invitrogen, Carlsbad, CA).

### Statistical analysis

Statistical analyses were done in JMP Pro 16 (SAS Institute Inc., Cary, NC, USA). Pen was considered the experimental unit (N = 12/experiment) and bird the observational unit (N = 36/experiment). Raw rTL data from all three experiments were z-transformed to allow for comparison between experiments [82]. Z scores were then analyzed using linear mixed models with environmental complexity, stocking density, and their interaction as fixed factors. Pen numbers nested within experiment and PCR plate number were included as random variables. The model was selected based on the research objective, determining the association between treatments and rTL outcomes (Z scores). Our mixed model accounted for repeated sampling from individuals within the experimental unit (pen), which matches the nature of the research design. Model assumptions were assessed using Levene’s test (variance homogeneity), normal quantile plots (normal distribution of data residuals), and by reviewing skewness and kurtosis outcomes. Model fit was assessed based on AICc and BIC values. The robustness of mixed-effects models allows their use even if the distributional assumptions are violated [83]. Post-hoc analysis was performed with Tukey HSD corrections. For validation of the qRT-PCR assay, a linear correlation was assessed with the TRF values as the response variable and the relative telomere lengths from qRT-PCR assays as the predictor. Associations were considered significant at P* ≤ *0.05 and a trend at P* ≤ *0.1. All data are presented as LSmeans of z-scores and error bars indicate 95% confidence intervals. Analysis was carried out following the minimum reporting recommendations for PCR-based telomere length measurements defined by the Telomere Research Network (Tulane, New Orleans, LA).

## Results

### Relative telomere length of Kidney and Gonad samples

**Fig 2:**
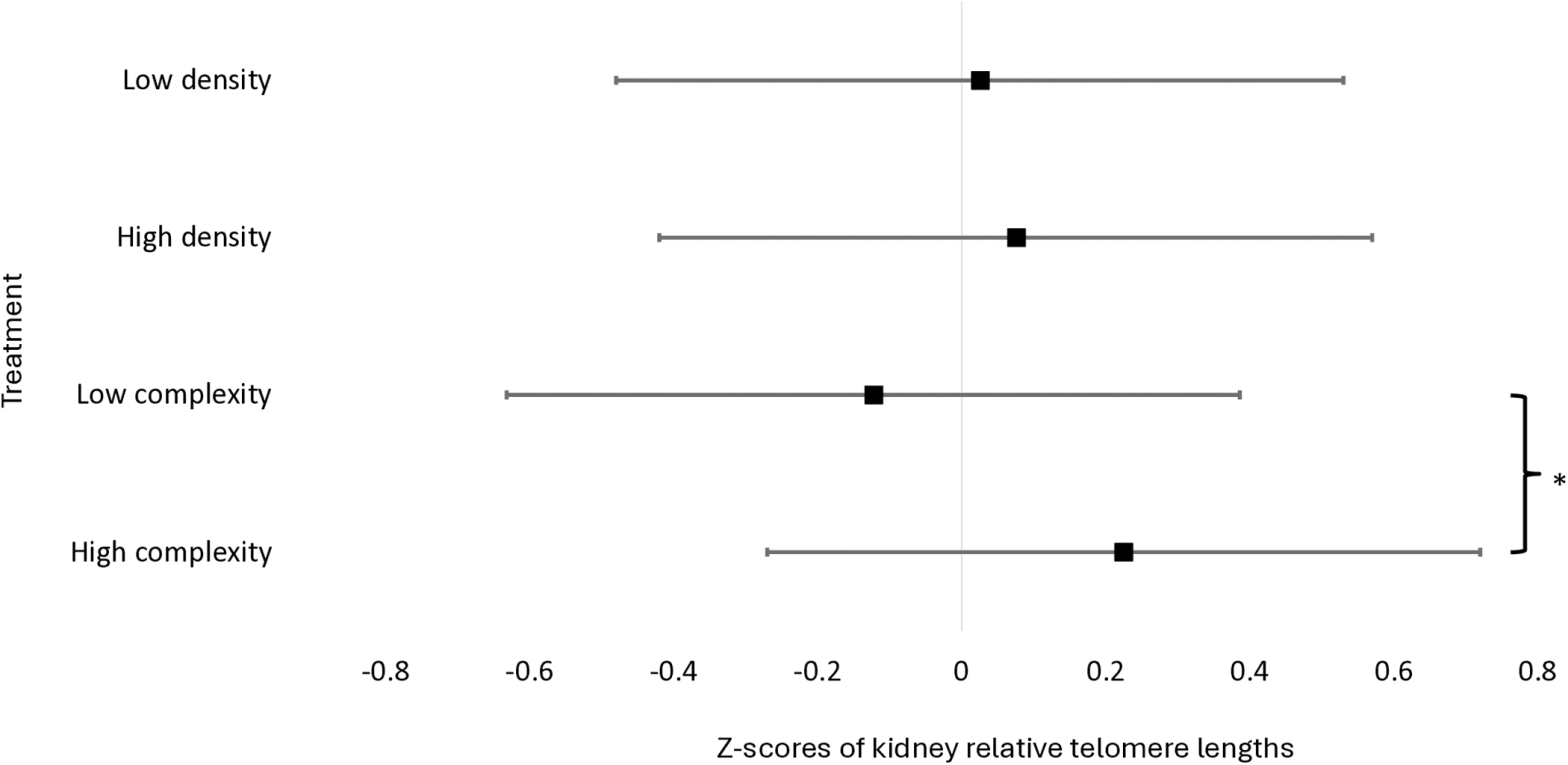
Z-transformed relative telomere lengths (rTL) from kidney samples collected from broilers at 48 days of age (N = 105). Data are presented as LSmeans (black square) with 95% confidence intervals (whiskers). * indicates a difference at P ≤ 0.05.

**Fig 3:**
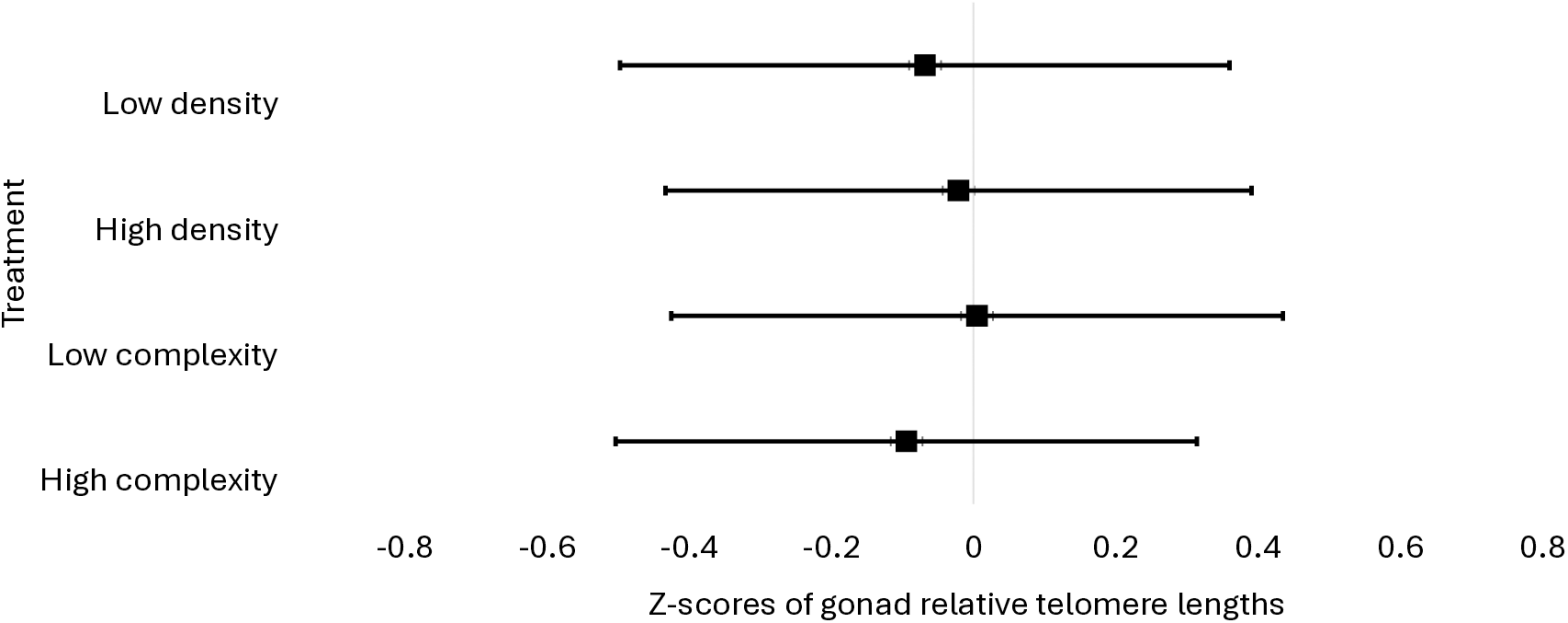
Z-transformed relative telomere length (rTL) measurements from gonad samples collected from broilers at 48 days of age (N = 108). Data are presented as LSmeans with 95% confidence intervals (whiskers).

Z-transformed kidney rTL scores tended to be higher (F_1,107_ = 4.17; P = 0.0503; S1 Table and S2 Table) in birds housed in high-complexity pens compared to low-complexity pens (Fig 2). Z-transformed kidney rTL scores were not impacted by stocking density (F_1,107_ = 0.08; P = 0.77). Z-transformed gonad rTL scores were not impacted by environmental complexity (F_1,107_ = 0.13; P = 0.71) or stocking density (F = 0.04; P = 0.84, Fig 3). The environmental complexity × stocking density interaction did not impact kidney (F_1,107_ = 0.49; P = 0.49) or gonad rTL (F_1,107_ = 0.09; P = 0.77). Means of untransformed rTL data from all three experiments are available in Table 1.

**Table 1:** Raw relative telomere length means for kidney and gonad samples from broilers at 48 days of age (N = 108/tissue), by treatment group.

Treatment	Kidney	Gonad
Low density	0.99	1.30
High density	1.03	1.33
Low complexity	0.91	1.34
High complexity	1.10	1.29

### Validation of qRT-PCR assay

**Fig 4:**
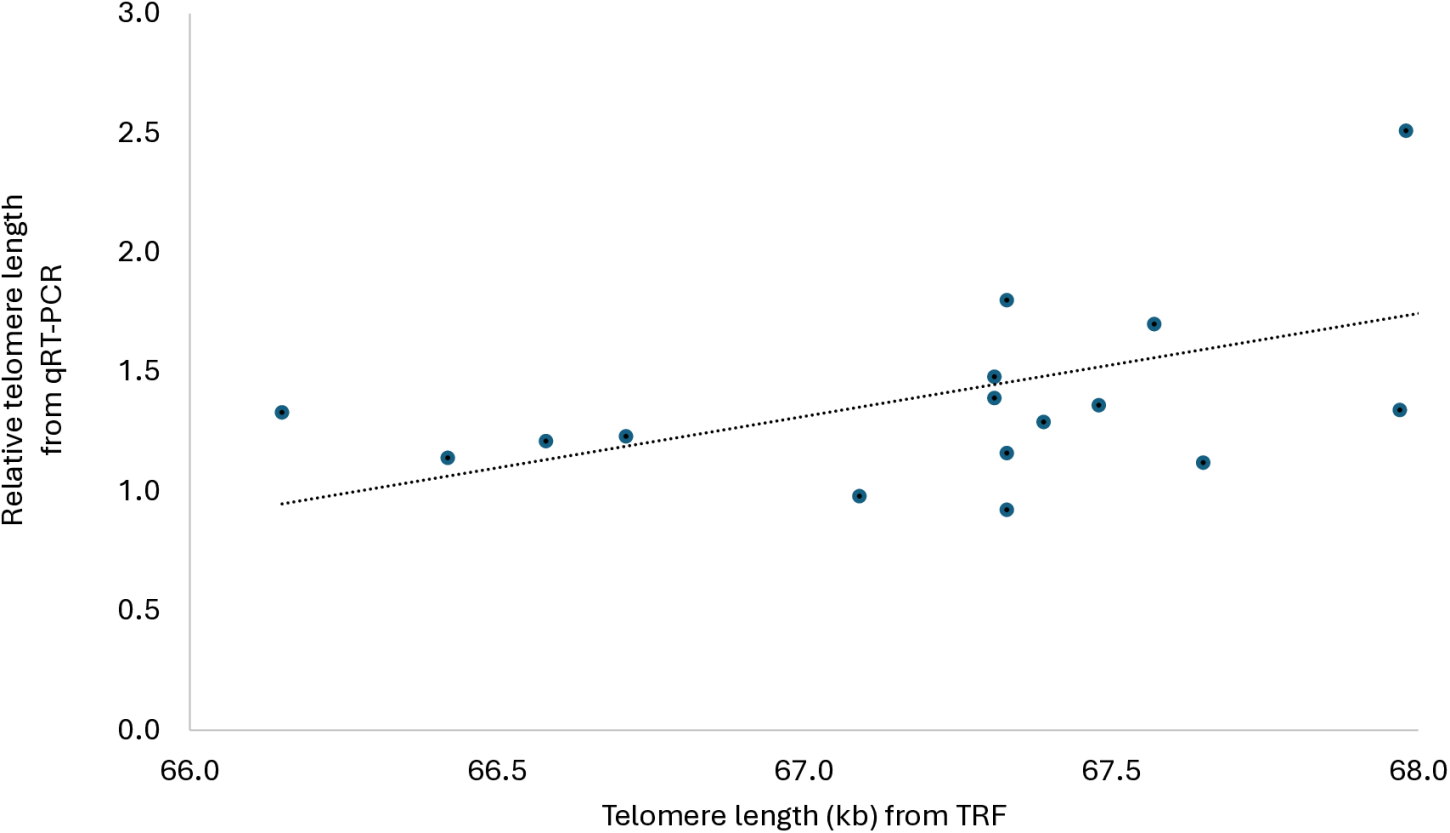
Linear correlation between raw relative telomere length data from qRT-PCR and telomere length data from TRF analysis using both gonad and kidney samples (N = 17).

The correlation between qRT-PCR and TRF analysis outcomes was positive, low (r^2^ = 0.28; *β* [95% CIs] = 0.529 [0.06,0.80]), and significant (P = 0.029; Fig 4). Mean (raw) telomere length through TRF analysis was 67.3 kb (min = 66.2 kb, max = 68.0 kb). Gonad samples tended to have longer telomeres than kidney samples when quantified via qRT-PCR (F_1,16_ = 3.61; P = 0.077) and had longer telomeres than kidney samples when quantified via TRF (67.6 vs. 67.0 kb; F_1,16_ = 11.06; P = 0.005).

## Discussion

We investigated the effects of environmental complexity and stocking density on telomere length as a measure of cumulative experience in broiler chickens. We observed a small but positive effect of environmental complexity on z-transformed kidney rTL scores across three replicate experiments. These results could indicate that rTL is sensitive to positive housing conditions and thus provides insights into broilers’ cumulative experience, although the effect was small. These results are the first indication that a complex environment can slow cellular aging in kidney tissue in broiler chickens, albeit with a small effect size. However, z-transformed kidney rTL scores were not sensitive to the negative impacts of high stocking density, indicating that rTL did not decrease due to negative housing conditions related to density, or that high stocking density did not negatively impact cumulative experience. The significant positive but low correlation between TRF and PCR assay outcomes indicates that the qRT-PCR approach is a valid method to assess rTL in broiler chicken DNA samples.

We found support for our hypothesis that birds housed in high-complexity pens show higher kidney rTL scores across all three trials. This implies that the birds housed in complex environments tended to show less telomeric damage than birds in environments with little complexity. This suggests that birds in complex environments had a somewhat more positive cumulative experience than birds in simple environments. These higher rTL scores in high-complexity birds are consistent with previous work in humans where individuals with a more positive psychological or physical well-being show longer telomeres [12,13,45]. People that were not suffering from anxiety disorders or depression had longer leukocyte telomeres than people with these disorders [12,13]. Healthy women showed slower TL decline when exposed to stressors compared to less healthy women [45]. Thoroughly reviewed by Zhang et al. [46], some TL data in livestock show similar trends, although some did not find TL associations with negative stressors or abnormal behaviors, such as a lack of association with abnormal oral behavior [84] or racing performance [85] in horses. Yet, healthy dairy cattle had longer telomeres than less healthy individuals, indicated by cull rates being lower in cows with long telomeres compared to cows with short telomeres [86]. Brahman cow parity was negatively associated with leukocyte TL [87]. The authors hypothesized that the differences in TL may originate from psychological and physiological stress of raising a calf [87]. In broilers, the provision of environmental enrichments to create a complex environment is considered to contribute to positive experience [6,58,59,61], and our results suggest that complex environments may promote physiological resilience in these birds. In line with our findings, control-group broiler chickens showed longer buffy coat TL compared to chickens that were fed a diet with corticosterone to induce chronic stress [49]. We consider two potential mechanisms for the higher rTL scores in high-complexity birds compared to low-complexity birds.

Firstly, positive experience in response to a complex environment may have reduced the number of distressing events experienced and provided opportunities for positive experiences. This decreased oxidative stress and telomeric DNA damage, reducing or mitigating telomere shortening due to distressing events. Chronic or acute stress was not assessed in this study; thus, the reduced distress in the high-complexity treatment cannot be confirmed. Yet, environmental complexity can decrease distress in birds [57,62,88–90], and chronic stress did reduce TL in broilers [49]. Aligned, broilers housed in high-complexity pens were less fearful following three acute stressors (sound stress, heath stress, and crating stress) compared to broilers housed in low-complexity environments [62]. Laying hens housed in high-complexity pens had a reduced startle reflex and comb temperature in response to acute stressors (light flash, restraint, and sudden appearance of a novel object) compared to low-complexity pens, indicating that those birds were more resistant to distress compared to birds from low-complexity environments [88]. Based on these previous findings, we infer that the small but positive impacts of high complexity on kidney telomere length are at least in part due to a reduction and mitigation of distressing situations.

Secondly, positive experience in response to a complex environment may have increased telomerase production in kidneys, repairing and lengthening telomeres. As telomerase production was not quantified in the current study, and no studies have investigated telomerase production in commercial poultry in response to housing conditions, this mechanism is not yet confirmed. These two mechanisms are not mutually exclusive but rather could have interacted to result in longer kidney telomeres in high-complexity birds compared to low-complexity birds. Future research should investigate how environmental complexity impacts telomerase production and distress responses in relation to telomere length to better determine how positive experience impacts kidney telomere length in broiler chickens.

Contrary to our hypothesis, stocking density did not negatively impact overall rTL values. These results somewhat contradict a previous study that showed that the telomere length in Ross 308 broilers was shorter in birds raised in high-density cages (0.058 m^2^/bird) compared to low-density cages (0.116 m^2^/bird, [20]). Space allowance in our experiments was approximately 0.08 m^2^/bird in high-density pens and 0.16 m^2^/bird in low-density pens. Thus, our high-density treatment provided birds with more space than the high-density treatment reported in [20] and our low-density treatment provided more space than the low-density treatment in the previous study. It is possible that the stocking density over 42 kg/m^2^ as tested in [20] caused excess distress compared to the high density in the current study, in turn leading to shortened telomeres, which could be an effect not reflected at the high density in the current study. Few production systems exceed stocking densities of 42 kg/m^2^ (0.081 m^2^/bird). For example, the United Kingdom Code of Practice allows a maximum stocking density of 39 kg/m^2^ with stringent requirements [91]. The European Council Directive (2007/43/EC) similarly restricts the maximum density to 42 kg/m^2^. In the United States no legislation limits the stocking density for broiler chicken production, however, 95 percent of broilers are produced by companies following the National Chicken Council Animal Welfare Guidelines, which recommends a maximum stocking density of 41.5 kg/m^2^ for broilers with target weights of 2.5 to 3.4 kg (NCC, 2020). In general, high stocking density is considered a negative stimulus which can lead to decreased foot and leg heath [51,54,55,92–94], and increased distress compared to low density [52,94]. However, there are studies that do not observe a negative impact of high density on broiler affective state [58,59] and distress [53]. For example, Ross 708 broilers raised in high-density pens showed decreased fear when compared to birds raised in low-density pens [59]. Maximum stocking density determines the number of birds placed based on birds’ average weight at the end of production. This means that stocking density is much lower in the early stages of production and density will increase as birds grow. Negative impacts of stocking density are observed at densities over 30 kg/m^2^ [94,95]. A stocking density of 30 kg/m^2^ was approximately reached in the high-density pens at d35 of age [96]. Therefore, it is possible that negative effects of high density only impacted the birds from d36-d50 of life. In this case, there may not be enough time for sufficient telomere shortening to occur and be detected. Additionally, stocking densities up to 42 kg/m^2^ may not create a sufficiently negative environment to cause damage to telomeric DNA. Therefore, stocking densities of 42 kg/m^2^ might not elicit negative cumulative experience in broilers in the experimental conditions tested or may not have a detectable impact on cumulative experience when measured with rTL values. We recommend further investigation into the impacts positive and negative stimuli on broiler chicken kidney telomere length to determine the viability of telomere length as a biomarker of cumulative experience in broilers. One negative stimulus that could be studied with prolonged ‘exposure’ could be feed restriction [97] in broiler or broiler breeder flocks.

We hypothesized that broiler gonadal rTL values would not be impacted by housing treatments, and results are aligned with this hypothesis. Gonadal tissue is primarily comprised of stem cells which produce high amounts of telomerase [74–76,79]. It was previously unclear whether tissues with high populations of stem cells are resistant to the impacts of cumulative experience, as no studies have investigated the impacts of cumulative experience on telomere length of these cell populations. The high concentrations of telomerase in gonadal stem cells repair any telomeric shortening that occurs due to cell division [75], but also likely repair damage caused by negative experiences. Thus, if low-complexity and high-density (assumed negative experiences) sped up biological gonadal cell aging, telomerase likely prevented or repaired the damage. Our study shows that gonadal rTL values are not impacted by positive or negative experiences associated with environmental complexity or stocking density in broiler chickens, as expected gonadal tissue may not show increased biological aging in response to cumulative experience. With few studies assessing TL in broilers, it is valuable to determine appropriate tissues when assessing the impact of stressors on cumulative experience. Finding that gonadal rTL is not impacted by the conditions tested in the current study confirms that these tissues should not be included when determining the impact of stressors on broiler chicken welfare. Therefore, studies should avoid use of these tissues (bone marrow, gonads) when investigating the impact of environmental conditions on telomere length.

Results did not support the hypothesis that birds from high-complexity/low-density environments would reduce telomere attrition (longer rTL) compared to birds from low-complexity/high-density environments. This is potentially due to low sample sizes for individual treatment combinations (N = 9 samples/treatment/experiment). It is possible that if sample sizes were increased, differences between treatments would have been observed. However, it is also possible that stocking densities of 42 kg/m^2^ did not impact telomere length in broiler chickens under experimental conditions. If true, no differences between interaction treatments would be expected as only environmental complexity would impact kidney telomere length, as is reflected in the findings. Another reason for a lack of measurable effect is that telomere dynamics may show too much inter-individual variation [98] or can be influenced by many physiological processes over time, making them a distant or indirect indicator of affective states, which are centrally regulated [99]. Affective states rely on neurological systems, thus mostly correspond to neurophysiological processes. This in turn can impact the physiology and behavior of the bird [99]. Although high stocking density has measurable effects on chicken behavior [100], the effect, if any, on rTL might be diluted by other factors influencing rTL. Future studies should investigate the interaction between stocking density and rTL at larger samples sizes or under commercial conditions to confirm the (lack of) impact of stocking density on telomere length. As rTL reflects individual experience, it would be valuable in future work to collect bird-level data on aspects of productivity and behavior to better understand these complex relationships.

Results from experiment 1 are impacted by an unintended continuous lighting period during the second week of life (day 8-day 14) and an outbreak of infectious bronchitis. Both increased light exposure [101] and pathogen response [102] can cause stress in broiler chickens which could negatively impact telomere length. If these adverse events severely decreased telomere lengths during experiment 1, it is possible that the combined telomere lengths during this experiment may also have been impacted. However, these were relatively short-term challenges (*<* 1 week) which may not have been long enough for significant telomeric damage to occur [63]. In line, gonad and kidney rTL levels were similar in all three experiments, indicating that increased light levels and infectious bronchitis likely did not severely impact telomere length at time of sampling.

The comparison of qRT-PCR and TRF assay outcomes confirmed a low, positive, and significant relationship between the two assay results which explained 28 percent of the variance. This correlation is lower than a previous study which compared qRT-PCR and TRF analysis [72]. Telomere quantification in chickens may be more susceptible to error due to their extremely long ‘mega-telomeres’, which may be more prone to mechanical sheering during analysis than shorter telomeric arrays [73]. The positive correlation between qRT-PCR and TRF can be increased by re-assaying the DNA samples via qRT-PCR and averaging the replicate rTL values from the two assays (25% increase in statistical power), or by extracting DNA on tissue samples twice and averaging the rTL values from the two extractions (17% increase in statistical power) [72]. We hypothesized that gonadal telomeres would be longer than kidney telomeres as gonads are composed primarily of stem cells which produce high concentrations of telomerase [74–76,79] Both qRT-PCR and TRF assays confirmed this hypothesis. Overall, these results show a positive relationship between qRT-PCR and TRF assay outcomes when quantifying telomere lengths in domestic chickens, rendering qRT-PCR a valid alternative to TRF analysis. This is the second experiment to investigate telomere length in broilers using qRT-PCR. Even with low sample sizes we were able to detect a small difference between complexity treatments. This suggests that telomere length quantification via qRT-PCR is promising as a high throughput and economically practical biomarker of cumulative experience in broiler chickens. Kidney sample collection is terminal, which is not desirable for an animal welfare biomarker. Determining rTL in blood would be a less invasive alternative, as used in [20]. However, organ tissue would be non-invasive and feasible when collected post-mortem at slaughter plants, as part of a retrospective welfare assessment in commercial flocks.

## Conclusion

Environmental complexity and stocking density did not impact gonadal telomere length of male broiler chickens. Following expectations, environmental complexity tended to positively impact kidney telomere length, indicating that the broilers raised in highly complex environments may have had a more positive cumulative experience and a lower biological age than broilers raised in low-complexity environments. Our results are the first to show the potential of telomere length as a measure of positive experience in broilers, as relative telomere length increased, or attrition was reduced in response to a positive environment. However, we were unable to confirm its potential as an indicator of the negative impact of high stocking density in broilers. Future studies should consider telomerase production and more prolonged negative stimuli when researching telomere length in broiler chickens. Finally, the qRT-PCR assay was validated as a method to quantify relative telomere length in broilers, although the agreement with TRF was low but significant.

## Supporting information

S1 Table Mixed model predictor output for z-transformed rTL data from kidney samples collected at 48 days of age (N = 105). (DOCX)

S2 Table Supplementary Table 2. Mixed model random effect output for z-transformed rTL data from kidney samples collected at 48 days of age (N = 105). (DOCX)
