# Supplementary material for: Telomere length as a biomarker for cumulative experience in broiler chickens
Source: PLoS One. 2025 Jun 25;20(6):e0326195. doi: 10.1371/journal.pone.0326195 (PMC12193831; doi:10.1371/journal.pone.0326195)
Supplement: S1 Table — (DOCX) [file pone.0326195.s001.docx]

**Supporting information for:**

**Telomere length as a biomarker for cumulative experience in broiler chickens**

| **Term** | **Estimate** | **Std Error** | **DFDen** | **t Ratio** | **Prob>\|t\|** | **95% Lower** | **95% Upper** |
| --- | --- | --- | --- | --- | --- | --- | --- |
| Intercept | -0.1476 | 0.2450 | 12.0 | -0.60 | 0.5581 | -0.681 | 0.386 |
| Stocking density [High-Low] | 0.0503 | 0.1730 | 30.1 | 0.29 | 0.7730 | -0.303 | 0.404 |
| Complexity [High-Low] | 0.3472 | 0.1698 | 28.3 | 2.04 | 0.0503 | -0.001 | 0.695 |

**Supplementary Table 1. Mixed model predictor output for z-transformed rTL data from kidney samples collected at 48 days of age (N=105).**
