# Supplementary material for: Telomere length as a biomarker for cumulative experience in broiler chickens
Source: PLoS One. 2025 Jun 25;20(6):e0326195. doi: 10.1371/journal.pone.0326195 (PMC12193831; doi:10.1371/journal.pone.0326195)
Supplement: S2 Table — (DOCX) [file pone.0326195.s002.docx]

**Supporting information for:**

**Telomere length as a biomarker for cumulative experience in broiler chickens**

**Supplementary Table 2. Mixed model random effect output for z-transformed rTL data from kidney samples collected at 48 days of age (N=105).**

| **Variance Component** | **Var Ratio** | **Estimate** | **Std Error** | **95% Lower** | **95% Upper** | **Wald p-Value** | **Pct of Total** |
| --- | --- | --- | --- | --- | --- | --- | --- |
| Pen ID [Experiment] | -0.0337 | -0.026 | 0.0799 | -0.1828 | 0.1307 | 0.7450 | 0.0 |
| PCR plate | 0.2976 | 0.2299 | 0.1606 | -0.0849 | 0.5447 | 0.1523 | 22.9 |
| Residual |  | 0.7727 | 0.1354 | 0.5634 | 1.1254 |  | 77.1 |
| Total |  | 1.0027 | 0.2124 | 0.6885 | 1.5947 |  | 100.0 |
